# The lamp of medicine of Ancient Egypt is still burning

**DOI:** 10.21542/gcsp.2020.16

**Published:** 2020-04-30

**Authors:** Ghazwan Butrous, Bradley Maron, Magdi Yacoub

**Affiliations:** 1 Professor of Cardiopulmonary Sciences; Medway School of Pharmacy University of Kent at Canterbury, UK; 2 Department of Medicine, Division of Cardiovascular Medicine, Brigham and Women’s Hospital, Boston, MA, USA; 3 Aswan Heart Center, Aswan, Egypt

In the land of Ancient Egypt, where the Pharaonic civilization flourished, one can encounter sophisticated methods of treating human illnesses. The Ancient Egyptians considered a disease and its treatment as a part of the divine intervention^1–3^. The Biblical text of the Passover story tells that the divine can send a series of ten plagues, most are diseases that affected various aged groups (Exodus 7:8-12:30; Psalm 77:42-51; 104:26-36). Not only were surgery and medicine practiced with care some 5000 years ago; the Pharaonic public health systems were innovative, providing water and removing waste in many places.

**Figure 1.**
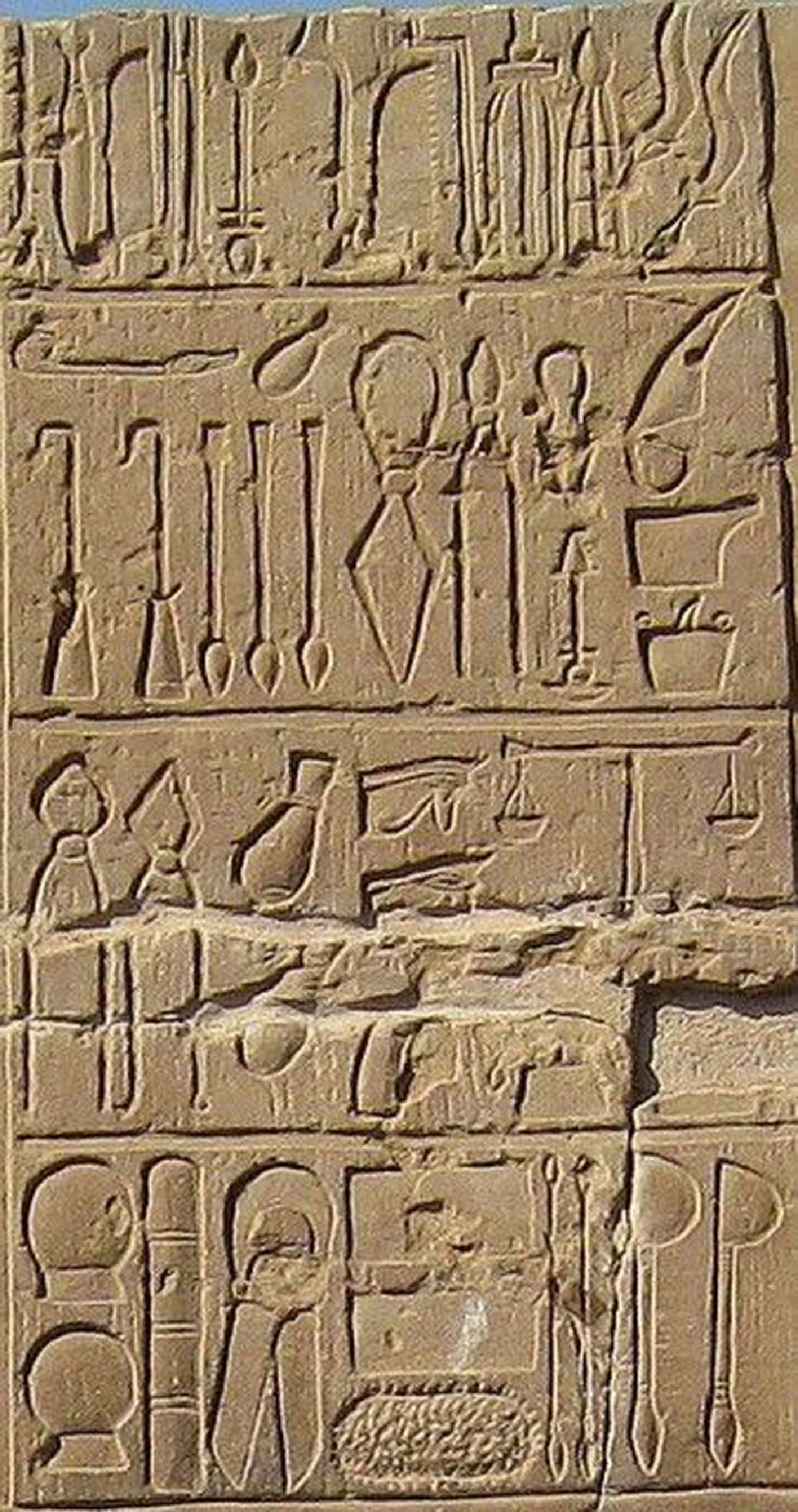
Carving in the temple at Kom Ombo showing some of the medical and surgical instruments like scalpels, forceps, bone saws, dental tools used by the Ancient Egyptians.

For historians of medicine, it was rewarding that the Ancient Egyptians wrote about the diseases and medications and how they should be used. Archaeological sources like the various papyrus dating back as far as 2900 BC, written in hieroglyphs, now in multiple libraries and museums worldwide, described various illnesses and their treatment. There are many examples of these “medical” papyrus, such as the Ebers papyrus, the Edwin manuscripts Smith Papyrus, the Hearst Papyrus, the London Medical Papyrus, and others^4^. The Ancient Egyptians also depicted many medical and health issues with writings and symbols in various tombs, temples, and other archaeological sites (Figure 1).

Our knowledge of Ancient Egyptian diseases has increased recently with the analysis of skeletal and mummified remains using modern imaging techniques like X-rays, computed tomographic imaging, magnetic resonance, lectron microscopes, mass spectrometry and forensic techniques that, collectively, provide a unique glimpse of the state of health in Egypt over 4000 years ago^5^. These advances also give us an idea about the spectrum of diseases Ancient Egyptians suffered: headache and emotional stress among tomb builders; various infectious diseases such as tuberculosis and worm infection; kidney stones; snake or scorpion bites; poliomyelitis; leprosy, and plague^6,7^.

Lung and heart disease among the ancients are of particular interest to modern-day investigators. Evidence of severe atherosclerotic vascular disease has been reported after careful examination of many mummies in various studies^9^. For example, one study of 44 mummies revealed that nearly half had evidence atherosclerosis^8–10^. Sandstorms, indoor cooking, metal working, mining, and stone carving all created a Mesopotamian form of ‘air pollution’ by virtue of whipped-up airborne particulates that could easily be inhaled^11,12^.

Indeed, obstructive lung diseases were documented in mummies by Eddie Tapp and others^11,13,14^. In a series of studies of the lungs of 15 mummies at the University of Manchester, Roger Montgomerie^15^, a doctoral student observed tiny microscopic particles in the lungs. One image in this thesis (page 136) showed deposits in the lung vasculature that were indicative of arterial pulmonary remodelling. Obstructive lung disease, in turn, is known to enhance pulmonary artery remodelling and pulmonary hypertension, as discussed in the paper by Michael McGettrick and Andrew Peacock in this issue^16^.

The other conjecture - that pulmonary vascular disease could have affected Ancient Egyptians – is partially verified by mummy examinations which have shown evidence of various worm infections^17^. An extensive autopsy of an Ancient Egyptian teenage male weaver, named Nakht, found that he was infected with many parasites: including *Schistosoma haematobium ,* and other worms besides malaria parasites like *Plasmodium falciparum.*

Schistosomiasis was prevalent in Ancient Egypt^18,19^, it is believed to have been spread to Egypt with the importation of monkeys and slaves during the reign of the fifth dynasty of Pharaohs (circa 2494–2345 BC).

Some Egyptologists suggested that this disease was mentioned 22 times in several Egyptian medical papyri available today from as early as 1500 years BC. Ancient Egyptians learned to avoid polluted water, and men who had regular contact with the river were advised to wear penile sheaths made of linen, as a protective measure^4,18^. Schistosomiasis remained endemic up to the 20^th^ century. In the 1920s, approximately 70% of the Egyptian male population was infected with *S. haematobium*.

Schistosomiasis is one of the common causes of pulmonary hypertension^19^ and might have been a condition that Ancient Egyptians suffered from, although pulmonary hypertension was not recognized as a disease until the early part of the 20^th^ century, as discussed in the review by Butrous in this issue^20^. Butrous emphasizes the importance of pulmonary hypertension as a global issue, and the diversity of this clinical condition varies from one country to another due the to the diversity of causes.

**Figure 2.**
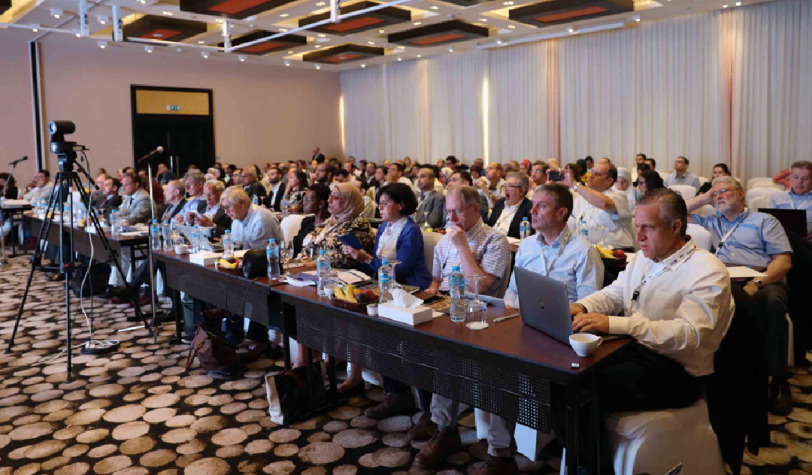
The first Pulmonary Hypertension of Aswan Heart Center Science & Practice Series Aswan, Egypt 18–19 October 2019.

Increasing interest and awareness of the condition emerged at the beginning of the 20th century, when the condition was first identified as a specific clinical disease by the international community. Thus, there was a necessity to communicate pulmonary hypertension to local physicians in collaboration with international experts. This was the drive for “The First Pulmonary Hypertension Aswan Heart Center Science & Practice Series”, held in Aswan, Egypt from 18–19 October 2019 under the auspices of the Magdi Yacoub Heart Foundation. There were 26 international speakers invited to participate in these two-day discussions on the recent advances and current practice in pulmonary hypertension. The meeting was attended by over 120 physicians and basic scientists from all over Egypt (Figure 2). A selection of the main presentations of this meeting is in this special issue of this journal.

Owing to increased awareness, the heterogeneity and complications of pulmonary hypertension are recognized to a greater extent and necessitated the importance of discussing deep phenotyping toward biomarker identification, as discussed by Paul Corris in this issue^21^.

The pulmonary hypertension pathobiology is not a clinical entity due to an abnormality of one specific pathway, signaling pathways, or mechanisms that regulate vascular remodeling and cardiopulmonary hemodynamic changes. It is is a syndrome formed from many causes and mechanisms. There are 5 classes of PH which reflect the pathogenetic background and histopathological appearance, each of them encompassing several subclasses. This was manifested in the ever-expanding range of cell types identified in the pathogenesis of pulmonary hypertension, presented in the concise summary of cellular and molecular changes in the lung by Bradley Maron^22^.

Current literature on the pathobiology of pulmonary hypertension revealed more complexity and new pathways. For example, in this issue, investigators from Kurt Stenmark lab^23^ discussed the role of the Complement System, which is at the core of innate immunity, playing a pivotal role in host homeostasis, inflammation, and defense against pathogens and unwanted host elements. They provide evidence of immunoglobulin-driven complement activation and the dysregulated complement activation in early pro-inflammation in the pathogenesis of hypoxia PH. In addition, the genetic and epigenetics components have been well recognized for now in the pathogenesis of pulmonary hypertension, adding to the complexity of the overall picture.

State of the art on this subject was reviewed by the Nicholas Morrell group in this issue^24^. They suggested that, besides the recognized mutation in some pathways, environmental factors may account for some of these idiopathic cases. It seems likely that an additional, unknown, rare genetic variation is responsible for many more examples which can influence the other abnormalities. Multi-omic analysis, genomics, and new bioinformatic tools, can provide insight into the causal drivers of pulmonary vascular disease - adding additional layers to the understanding of the disease pathobiology - and are likely to enter clinical settings in the near future.

The complex pathogenesis leads many investigators to consider microenvironmental inflammation and its cross-talk with vascular cells as the major underlying pathogenic pathway to this condition - suggesting some similarities with cancer as discussed in this issue^25^. These authors also discussed the thesis that pulmonary vascular abnormalities may thus contribute to symptoms presented by lung cancer patients.

This multifactorial and diverse pathobiology of pulmonary hypertension will influence the management and the approach to treatment, suggesting a need for more accurate and precise diagnostic methods and careful hemodynamic assessment with right heart catheterization as discussed by Stefano Ghio^26^. Clinicians carrying out the examination must be guided not only by technical recommendations, but also by accurate knowledge of the different clinical issues, including thorough risk stratification as summarized by Paul Corris^27^.

Recent clinical trials and evidence-based medicine supports the use of multidrug therapy rather than monotherapy in most pulmonary arterial hypertension patients, as discussed by Bradley Maron in his article “Pulmonary Arterial Hypertension: Rationale for Using Multiple vs Single Drug Therapy”^28^.

The last five years have seen an increasing interest in selected groups of pulmonary hypertension - mainly those for the chronic thrombotic diseases - after the approval of a new medical therapy to treat chronic thromboembolic pulmonary hypertension (CTEPH), and the advances in the pulmonary thromboendarterectomy. In this issue, Mario Gerges and Magdi Yacoub summarize the recent state-of-the-art with CTEPH, covering epidemiology, clinical presentations, and management strategy, including recent advances in the interventional treatment with balloon pulmonary angioplasty^29^. The authors also summarize the most recent 6th World Symposium on PH Task Force on CTEPH where a new CTEPH treatment algorithm was proposed.

The meeting was concluded with a case presentation of pulmonary hypertension in secondary antiphospholipid syndrome associated with systemic lupus erythematosus, once again showing the complexity and the extreme heterogeneity of the disease pathobiology^30^.

**Figure 3.**
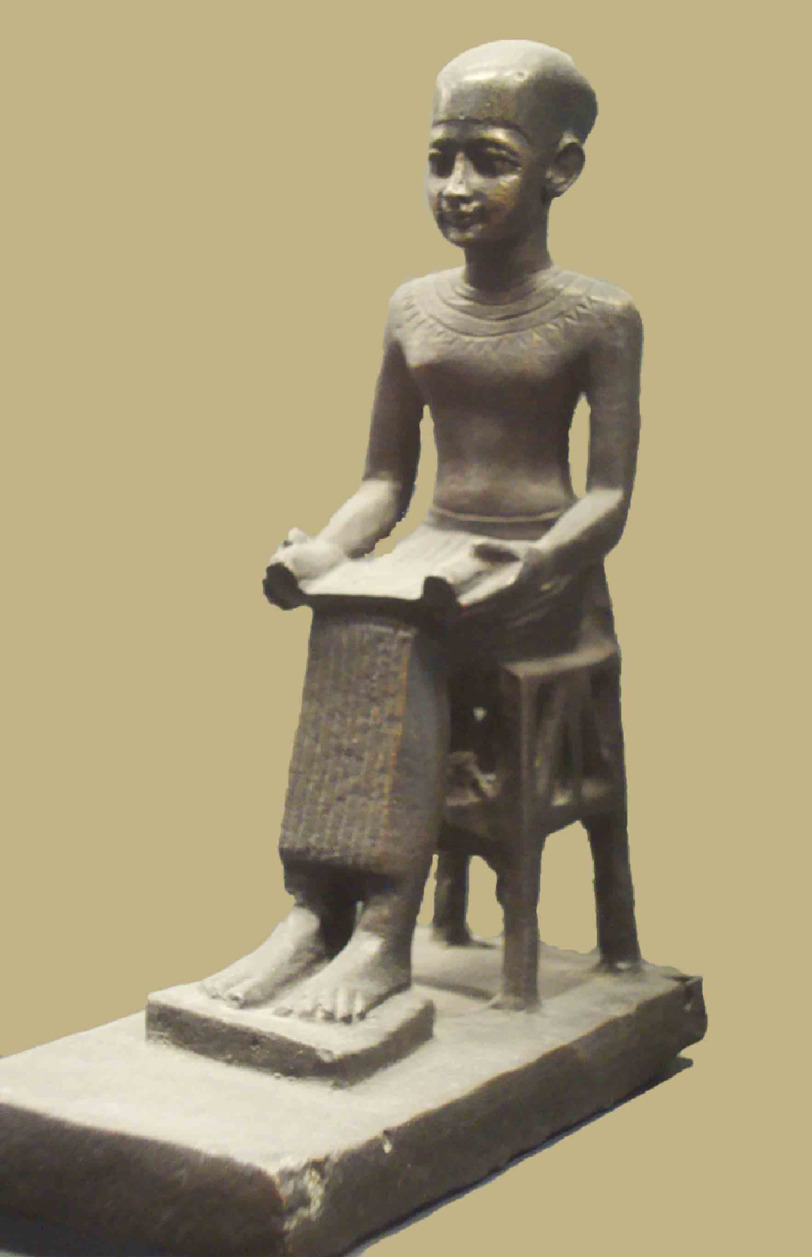
Statuette of Imhotep in the Louvre. He was depicted with an open scroll, probably communicating his wisdom.

The meeting was interactive. All participants and faculty had ample time for discussion and exchange of ideas, reflecting the intense spirit of communication that we learned from the Ancient Egyptians, who left us with many sources to discover their civilization. Archaeological data suggested the men of medicine, who were also sources of communication to the court and perhaps the public, were respected among Ancient Egyptians, as demonstrated in the famous statuette of the wise man and physician “Imhotep”^31^ - advisor to King Djoser, in the mid-27th century BC (Figure 3). Imhotep was a physician who extracted medicines from plants and was credited with the diagnosis and treatment of over 200 medical conditions. He was deified in the 7th century BC and revered as a medical demigod during many periods of the Pharaonic history. He was equated with the Greek god of Medicine, Asclepius. It was not surprising that Sir William Osler called him “*the first figure of a physician to stand out from the mists of antiquity*”. Imhotep was the protector of scholars and doctors and commonly presented sitting, with an open papyrus scroll, probably giving a talk or lecture! (Figure 3).

Ironically, his name was also mentioned in the inscriptions of Famine Stela, near Aswan, where the meeting was held. These relics reminded us of the history of the land which provided some of the earliest written evidence of medical practice.
